# Etanercept attenuates traumatic brain injury in rats by reducing early microglial expression of tumor necrosis factor-α

**DOI:** 10.1186/1471-2202-14-33

**Published:** 2013-03-15

**Authors:** Chung-Ching Chio, Chin-Hong Chang, Che-Chuan Wang, Chong-Un Cheong, Chien-Ming Chao, Bor-Chih Cheng, Chung-Zhing Yang, Ching-Ping Chang

**Affiliations:** 1 Department of Surgery, Chi Mei Medical Center, Tainan, Taiwan; 2 Department of Intensive Care Medicine, Chi-Mei Medical Center, Liouying, Tainan, Taiwan; 3 Department of Surgery and Department of Intensive Care Medicine, Chi-Mei Medical Center, Liouying, Tainan, Taiwan; 4 Department of Biotechnology, Southern Taiwan University of Science and Technology, Tainan, Taiwan; 5 The PhD Program for Cancer Biology and Drug Discovery, College of Medical Science and Technology, Taipei Medical University, Taipei, Taiwan

**Keywords:** Traumatic brain injury, Microglia, Tumor necrosis factor-alpha, Astrocyte, Neuron

## Abstract

**Background:**

Tumor necrosis factor-alpha (TNF-α) is elevated early in injured brain after traumatic brain injury (TBI), in humans and in animals. Etanercept (a TNF-α antagonist with anti-inflammatory effects) attenuates TBI in rats by reducing both microglial and astrocytic activation and increased serum levels of TNF-α. However, it is not known whether etanercept improves outcomes of TBI by attenuating microglia-associated, astrocytes-associated, and/or neurons-associated TNF-α expression in ischemic brain. A well clinically relevant rat model, where a lateral fluid percussion is combined with systemic administration of etanercept immediately after TBI, was used. The neurological severity score and motor function was measured on all rats preinjury and on day 3 after etanercept administration. At the same time, the neuronal and glial production of TNF-α was measured by Immunofluorescence staining. In addition, TNFα contents of ischemic cerebral homogenates was measured using commercial enzyme-linked immunosorbent assay kits.

**Results:**

In addition to inducing brain ischemia as well as neurological and motor deficits, TBI caused significantly higher numbers of microglia-TNF-α double positive cells, but not neurons-TNF-α or astrocytes-TNF-α double positive cells in the injured brain areas than did the sham operated controls, when evaluated 3 days after TBI. The TBI-induced cerebral ischemia, neurological motor deficits, and increased numbers of microglia-TNF-α double positive cells and increased TNF-α levels in the injured brain were all significantly attenuated by etanercept therapy.

**Conclusion:**

This finding indicates that early microglia overproduction of TNF-α in the injured brain region after TBI contributes to cerebral ischemia and neurological motor deficits, which can be attenuated by etanercept therapy. Studies in this model could provide insight into the mechanisms underlying neurological motor disturbance in brain-injured patients.

## Background

Tumor necrosis factor-alpha (TNF-α) is elevated in both the serum and cerebrospinal fluid of humans after traumatic brain injury (TBI) [1,2]. It is also elevated 1 to 4 hours after injury in clinically relevant models of TBI such as lateral fluid-percussion injury (FPI) [3-5]. The overproduction of TNF-α caused by TBI can be significantly attenuated by blockade of TNF-α synthesis or activity [6-8]. However, the cellular sources of this early elevation of TNF-α remain unclear. Evidence has indicated that TNF-α is predominantly expressed by neurons [7] or microglia [9,10] in the injured brain.

Etanercept is a TNF antagonist with anti-inflammatory effects. Systemic administration at the dosage of 5 mg/kg of body weight improves outcomes of TBI in rats by reducing microglial and astrocytic activation and activated inflammation (e.g., increased serum levels of TNF-α) [8]. Again, it is not known whether etanercept improves outcomes of TBI in rats by reducing microglia-associated, astrocytes-associated and/or neurons-associated TNF-α expression in ischemic brain.

We explored these two questions by studying the interrelationship between the activation of microglia-TNF-α, astrocyte-TNF-α, and neuron-TNF-α double labeling cells in the ischemic brain areas and the development of cerebral ischemia and neurological and motor dysfunction as well as the attenuating of cerebral ischemia and neurological and motor dysfunction by etanercept therapy after TBI.

## Methods

### Animals

Male Sprague–Dawley rats (280–300 g), obtained from the Animal Resource Center of the Taiwan National Science Council, were housed four per group at an ambient temperature of 22 ± 1°C with a 12-h light–dark cycle. Pelleted rat chow and tap water were available *ad libitum*. All protocols, designed to minimize discomfort in the animals during surgery and in the recovery period, were approved by the Institutional Animal Care and Use Committee of Southern Taiwan University of Science and Technology.

### Surgery

The rats were anesthetized with sodium pentobarbital (25 mg/kg, intraperitoneally [i.p.]; Sigma Chemical, St. Louis, MO, USA) and a mixture containing ketamine (4.4 mg/ kg, intramuscularly [i.m.]; Nankuang Pharmaceutical, Tainan, Taiwan), atropine (0.02633 mg/kg, [i.m.]; Sintong Chemical, Taoyuan City, Taiwan), and xylazine (6.77 mg/kg, [i.m.]) (Bayer AG, Leverkusen, Germany). Both the femoral artery and vein on the right side were cannulated with PE50 polyethylene tubing to monitor blood pressure and analyze blood gas respectively. After cannulation, the wound was sutured and the rats were placed in a stereotaxic frame. Each rat’s scalp was sagittally incised, and then the rat was subjected to a FPI [11,12]. Prior to FPI, a 4.8-mm circular craniotomy was performed midway between the lambda and bregma skull points 3.0 mm to the right of the central suture. A modified Luer-Lock connector (trauma cannula) (2.6 mm inner diameter) was secured into the craniotomy with cyanoacrylate adhesive and dental acrylic. Subsequently, the FPI was induced by a pressure (severe intensity: amplitude of 2.2 atm ospheres) delivered by a fluid percussion device (Virginia Commonwealth University Biochemical Engineering, Richmond, VA, USA). The rat was removed from the device with the acrylic removed, and the incision sutured. Each injured and sham-injured animal for the FPI model was housed individually and closely evaluated for behavioral recovery immediately after the FPI. During the surgical procedure, mean arterial pressure, heart rate, and core temperature were continuously monitored for indicating depth of anesthesia.

### Experimental procedures

The rats (n=96) were randomly allocated into the following groups: 1) FPI+saline control group (n=32): rats were subjected to FPI plus a dose of normal saline (1 ml/kg body weight [i.p.]) once every 12 h for 3 consecutive days [8]; 2) FPI+etanercept group (n = 32): this group was subjected to FPI plus etanercept (5 mg/kg body weight [i.p.]) once per 12 h for 3 consecutive days; and 3) untreated Sham group (n = 32): rats were subjected to the same surgical procedures as the other two groups, but were not given a FPI. The injection volume was the same for both saline and etanercept groups. The sham-group was untreated.

In experiment 1, etanercept (5 mg/kg [i.p.]) (n=8) or saline (1 ml/kg [i.p.]) (n=8) was randomly administrated immediately after FPI, and their effects on neurological motor performance were assessed preinjury and on 4 days post-FPI. Another 8 untreated sham-operated rats were used as controls. Etanercept (ENBREL™ Wyeth Laboratories, Hampshire, UK) was reconstituted with normal saline according to the manufacturer’s instructions.

In experiment 2, three days after FPI, immediately following the neurological motor tests, all the rats used in experimental 1 were killed 3 days after FPI for brain contusion assessment.

In experiment 3, etanercept (5 mg/kg [i.p.] (n=8) or saline (1 ml/kg [i.p.] (n=8) was randomly given to each rat immediately after FPI once every 12 h for 3 consecutive days, and the effect on the number of the co-localization of TNF-α and microglia specific marker cells, TNF-α and neurons specific marker cells, and TNF-α and astrocyte specific marker cells in the cortex, white matter, hippocampus, or hypothalamus was determined. Untreated sham group, FPI+saline group, and FPI-etanercept group were killed 72 h post-FPI (n=8 for each group) for immunohistological evaluation.

In experiment 4, untreated sham group (n=8), FPI+saline group (n=8), and FPI+etanercept group (n=8) animals were killed 72 h post-FPI for determination of TNF-α protein level in ischemic brains.

### Neurological function evaluation

Acute neurological injury was assessed in all rats on the day prior to and on 3 days after surgery using a neurological severity score (NSS) [13]. NSS is a composite of the motor (muscle status, abnormal movement), sensory (visual, tactile and proprioceptive) and reflex tests. One point was given for failure to perform a task. Thus, the higher score, the more severe is injury, with a maximum of 14 points. The NSS was measured on all rats pre injury and on day 3 after etanercept administration.

The inclined plane was used to measure limb strength [14]. The rat was placed, facing right and then left, perpendicular to the slope of a 20×20-cm ruffed surface of an inclined plane starting at an angle of 55°. The angle was increased or decreased in 5° increments to determine the maximal angle at which a rat could hold to the plane. The data for each day were the mean of the left- and right-side maximal angles. All behavioral tests were examined and independently scored by two observers who were unaware of what treatment the rats had been given. These scores were averaged to arrive at one score for each rat for the behavioral session. The rats were tested pre injury and on day 3 after etanercept administration.

### TNF-α content of ischemic cerebral homogenate

Cerebral hemispheres were quickly dissected free and kept on ice in physiological salt solution (mM: NaCl 119; KCl 4.7; MgSO4 1.17; KH2PO4 1.18; NaHCO_3_ 25; EDTA 0.026; and CaCl2 2.5) containing 5 mM glucose. Segments of cerebral cortex (75 mg i.e., approximately the weight of each cerebral hemisphere) were weighted, cut into small pieces, dispersed by aspiration into a pipette and suspended in 1 ml of physiological salt solution in a test tube. Samples were kept on wet ice for 20 min before use. The homogenates were centrifuged at 7,500 rpm (5,150×g) for 20 min. The supernatants were used for measuring TNF-α concentrations. TNF-α concentrations were measured using commercial enzyme-linked immunosorbent assay (ELISA) kits (Biosource International Inc. Boshide Company, Wuhan, China) and following the manufacturer’s instructions. The minimum detectable concentrations of TNF-α were 1.1 pg/ml. There was no cross-reactivity reported with other cytokines. All samples were assayed is duplicate.

### Cerebral ischemia assay

The triphenyltetrazolium chloride (TTC) staining procedures are described elsewhere [15]. Three days after they had undergone FPI, all the rats were deeply anesthetized (sodium pentobarbital, 100 mg/kg [i.p.]) and then perfused intracardially with saline. Their brain tissue was then removed, immersed in cold saline for 5 min, and sliced into 2.0-mm sections with a tissue slicer. The brain slices were incubated in 2% TTC dissolved in phosphate buffer saline (PBS) for 30 min at 37°C and then transferred to 5% formaldehyde solution for fixation. The volume of ischemia, revealed by negative TTC stains that indicated dehydrogenase-deficient tissue, was measured in each slice and summed using computerized planimetry (PC-based Image Tools Software). The volume of ischemia was calculated as 2 mm (thickness of the slice)×[sum of the ischemia area in all brain slices (mm)^2^].

### Immunohistological determination

Serial 50-μm sections corresponding to coronal coordinates 0.8 mm to 5.3 mm posterior to the bregma were incubated in 2 mol/L HCL for 30 min, rinsed in 0.1 mol/L boric acid (pH 8.5) for 3 min at room temperature and then incubated with primary antibodies in phosphate-buffered saline (PBS) containing 0.5% normal bovine serum at 4°C overnight; secondary antibodies-incubated for 1 h at room temperature. The antibodies therein were, sequentially, mouse monoclonal anti-NeuN (Abcan, 1:200), mouse monoclonal anti-GFAP antibody (Abcam, 1:200), mouse monoclonal anti-Iba-1 antibody (Abcam, 1:200), goat polyclonal anti-TNF-α antibody (Santa Cruz, 1:50), DyLight 488-conjugated donkey-anti-goat IgG antibody (Abcam, 1:400), and Alexa Fluro 568-conjugated donkey anti-mouse IgG antibody (Invitrogen, 1:400). The sections were coverslipped with the mounting medium (fluorescent mounting medium; Dako). The labeled cells were calculated in 5 coronal sections from each rat and expressed as the mean number of cells per section. For negative controls sections, all the procedures were without the primary antibody. Primary and secondary antibodies for multiple-staining are listed in Table 1.

**Table 1:** Antibodies used for Immunofluorescence staining

**Antibody**	**Antigen**	**Host**	**Company**	**Catalog#**	**Dilution**
Primary antibody					
NeuN	neuron	mouse	Abcam	ab104224	1:200
GFAP	astrocyte	mouse	Abcam	ab4648	1:200
Iba-1	microglia	mouse	Abcam	ab15690	1:200
TNF-α	TNF-α	goat	Santa Cruz	SC-1351	1:50
Secondary antibody (conjugation)					
Mouse IgG (Alexa Fluor 568)	Mouse IgG	goat	Invitrogen	A11031	1:400
Goat-IgG (DyLight 488)	Goat IgG	donkey	Abcam	ab96931	1:400

Images of the fluorescent immunohistochemistry for immune cells were captured at 100x magnification using a fluorescence microscope system (Zeiss Axiovision; Zeiss Gmbh, Göttingen, Germany), and images from bregma −0.8, -1.5, -2.5, -3.0, and −3.5 mm from each animal were evaluated. In each image, immune-positive cells showing staining with a cellular morphology and above background level were manually and exhaustively counted using the Axiovision image analysis software (Zeiss Gmbh), All cell counts were performed by an investigator (C. P.C.) blinded to the treatment status of each animal.

### Statistical analysis

The data are presented as mean ± standard deviation (SD). A repeated measures analysis of variance was used to test the treatment-by-time interactions and the effect of treatment over time on each score. Duncan’s multiple range test was used for *post hoc* multiple comparisons among means. Analyses for all behavioral variables used Student’s unpaired t-test to compare variables between groups. Bonferroni’s analysis was then performed when appropriate, to determine post-hoc significance at individual time point. Data was analyzed using Statistica, Software® and, in all cases, statistical significance was set at P<0.05.

## Results

### Acute effects of FPI

The average intensity of the fluid pulse delivered to animals in the injured group was 2.24±0.05 atm (mean±SEM). Immediately following this impact, all rats experienced a period of apnea (lasting approximately 25 sec), hypertension (approximately up to ~140 mmHg and lasting ~25 sec), and tachycardia (~390 beats/min and lasting more than 120 minutes). Sham-injured animals showed no apnea, hypertension, or tachycardia. There was no difference between 2 treatment groups.

### FPI caused neurological and motor dysfunction, which etanercept attenuated

**Figure 1:**
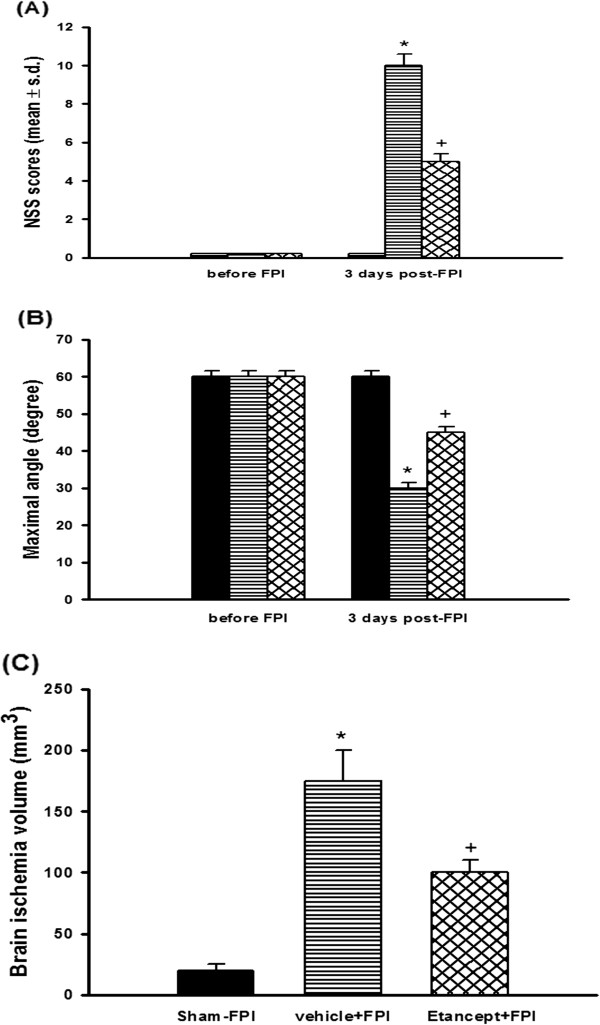
**Etancercept attenuated FPI**-**induced increased neurological severity scores** (**NSS**) **(A), decreased motor performance (B), and increased brain ischemic volume (C).** *The FPI+saline group (▤; n=8) showed a significant increase in NSS (P<0.01), a significant decrease in maximal angle (P<0.05), and a significant increase in brain ischemic volume (P<0.05) compared with the untreated sham-operated group (■) 3 days post-FPI. ^+^The FPI+etanercept group (▩; n=8) showed a significant decrease in NSS (P<0.05), a significant increase in maximal angle (P<0.05), and a significant decrease in brain ischemia volume (P<0.05) compared with the FPI+saline group (▤; n=8).

Three days after the rats had been subjected to FPI, behavioral tests revealed that the NSS of both the (FPI+saline) group and the (FPI+etanercept) group were significantly (P<0.05) higher than those of the untreated sham-FPI group (10 or 5 vs 0; n=8 for each) (Figure 1A). However, compared with those of the (FPI+saline) group, the the NSS values of the (FPI+etanercept) group (n=8) were significantly (P<0.05) lower. In contrast, motor function tests showed that the maximal angles of the (FPI+saline) group were significantly (P<0.05) lower than those of the sham-FPI group (60° Vs 30°; n=8 for each) (Figure 1B). Compared with those of the (FPI+saline) group, the maximal degrees were significantly (P<0.05) higher in the (FPI+etanercept) group (30° Vs 45°; n=8 for each) (Figure 1B).

### FPI induced cerebral ischemia, which etanercept attenuated

TTC staining showed that the (FPI+saline) group had significantly (P<0.001) larger areas of brain ischemia than did the sham-FPI group (Figure 1C). (186±26 mm^3^ vs 21±5 mm^3^; n=8 for each group). The cerebral ischemia areas were significantly (P<0.01) smaller in the FPI+etanercept group than in the FPI+saline group (104±12 mm^3^ vs 186±26 mm^3^; n=8 for each group) (Figure 1C).

### FPI caused the microglial production of TNF-α, which etanercept attenuated

**Figure 2:**
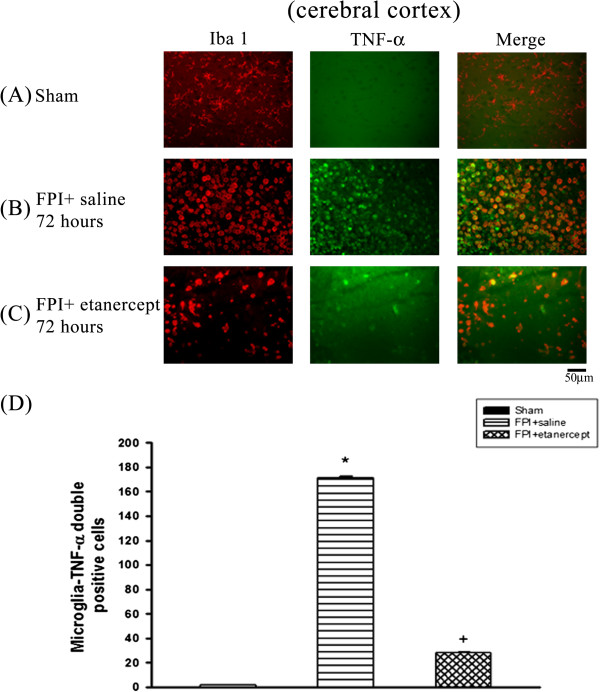
**Etanercept decreased FPI**-**induced increases in the number of co**-**localization of microglia and TNF-α specific marker cells in ischemic cortical regions.** Representative panel staining 72 h after sham operation or FPI, respectively for (**A**) a sham operation rat, (**B**) a FPI+saline rat and (**C**) a FPI+etanercept rat. (**D**) The FPI+saline group (▤) showed a significant increase in the number of co-localization of microglia and TNF-α specific marker cells in the ischemic cortex 72 h after FPI compared with the sham controls (■) (^*^P < 0.01). The FPI+etanercept group showed a significant decrease in the number of co-localization of microglia and TNF-α specific marker cells (▩) compared with the FPI+saline group (▤) (^+^*P* < 0.01). Each column and bar is the mean ± SD of eight rats per group.

**Figure 3:**
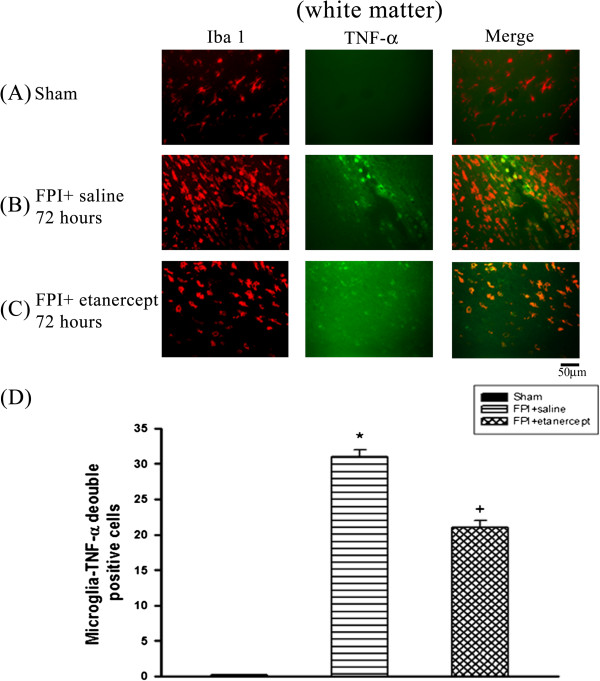
**Etanercept decreased FPI**-**induced increases in the number of co**-**localization of microglia and TNF-α specific marker cells in ischemic white matter regions.** Representative panel staining 72 h after FPI, respectively, for (**A**) a sham operation rat, (**B**) a FPI+saline rat and (**C**) a FPI+etanercept rat. (**D**) The FPI+saline group (▤) showed a significant increase in the number of co-localization of microglia and TNF-α specific marker cells in the ischemic white matter 72 h after FPI compared with the Sham controls (■) (*P < 0.01). The FPI+etanercept group (▩) showed a significant decrease in the number of co-localization of microglia and TNF-α specific marker cells in the ischemic white matter compared with the FPI+saline group (▤) (^+^*P* < 0.01). Each column and bar is the mean ± SD of eight rats per group.

**Figure 4:**
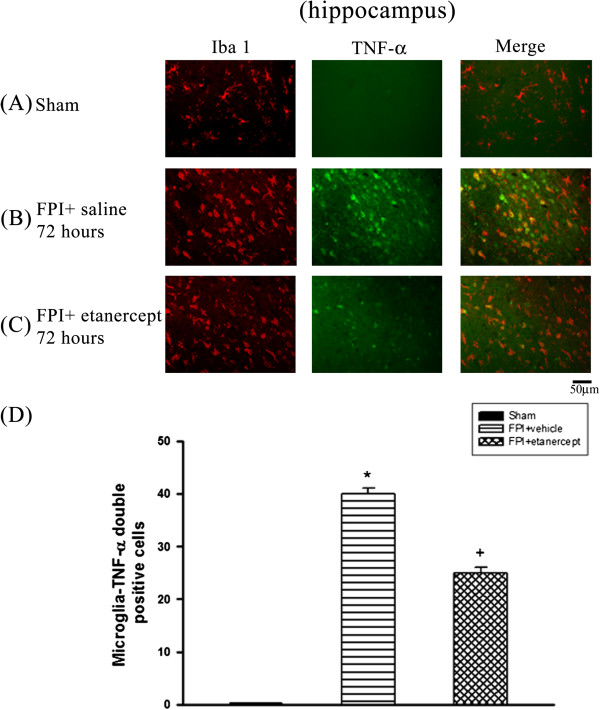
**Etanercept decreased FPI**-**induced increases in the number of co**-**localization of microglia and TNF-α specific marker cells in ischemic hippocampal regions.** The FPI+saline group (▤) showed a significant increase of the numbers of co-localization of microglia and TNF-α specific marker cells cells in the ischemic hippocampus 72 h after FPI compared with the Sham controls (■) (*P < 0.01). Representative panel staining 72 h after FPI, respectively, for (**A**) a sham operation rat, (**B**) a FPI+saline rat and (**C**) a FPI+etanercept rat. (**D**) The FPI+etanercept group (▩) showed a significant decrease in the number of co-localization of microglia and TNF-α specific marker cells in ischemic hippocampus compared with the FPI+saline group (▤) (^+^*P* < 0.01). Each column and bar is the mean ± SD of eight rats per group.

**Figure 5:**
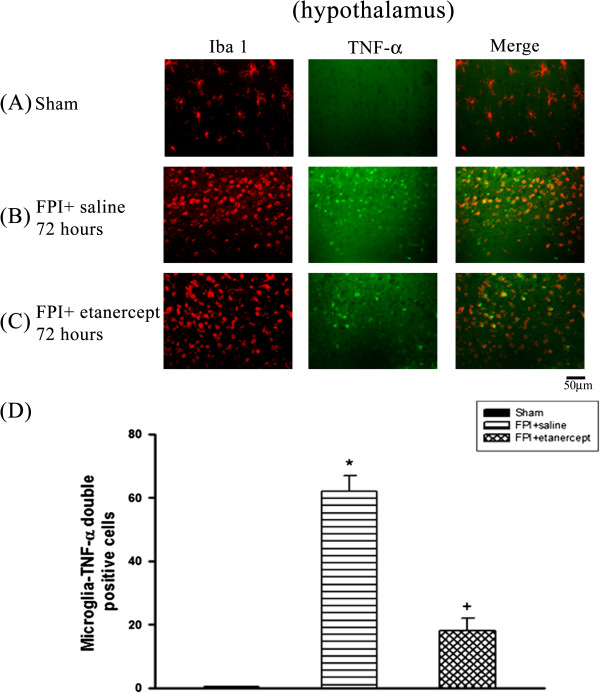
**Etanercept decreased FPI**-**induced increases in the number of co**-**localization of microglia and TNF-α specific marker cells in ischemic hypothalamic regions.** Representative panel staining at 72 h after FPI, respectively, (**A**) a sham operation rat, (**B**) for a FPI+saline rat and (**C**) a FPI+etanercept rat (**D**) The TBI+saline group (▤) showed a significant increase of the numbers of co-localization of microglia and TNF-α specific marker cells cells in the ischemic hypothalamus 72 h after TBI compared with the sham controls (■) (^*^P < 0.01). The FPI+etanercept group (▩ ) showed a significant decrease of the numbers of co-localization of microglia and TNF-α specific marker cells compared with the FPI+saline group (▤) (^+^*P* < 0.01). Each column and bar is the mean ± SD of eight rats per group.

Immunofluorescence staining revealed that the number of colocalization of microglia and TNF-α specific markers in the ischemic cortex (Figure 2), white matter (Figure 3), and hippocampus (Figure 4) and hypothalamus (Figure 5) were significantly higher (P<0.01) in the FPI+saline group than in the sham group, when evaluated 72 h after the start of FPI. Nevertheless, compared with those of the saline-treated FPI group, the etanercept-treated FPI rats had significantly (P<0.01) lower values of the numbers of co-localization of microglia and TNF-α specific markers in the ischemic cortex (Figure 2), white mater (Figure 3), hippocampus (Figure 4), and hypothalamus (Figure 5).

**Figure 6:**
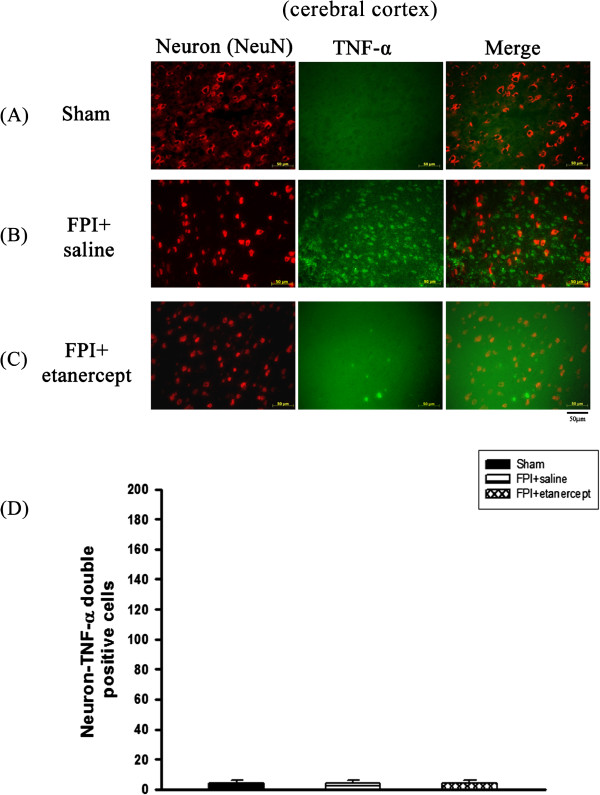
**Etanercept or FPI treatment did not affect the number of co**-**localization of neurons and TNF-α specific marker cells in the ischemic cerebral cortical regions.** Representative panel staining 72 h after sham operation or FPI, respectively for (**A**) a sham operation rat, (**B**) a FPI+saline rat and (**C**) a FPI+etanercept rat. (**D**) The FPI+saline group (▤) or the FPI+etanercept group (▩) showed an insignificant change in the number of co-localization of neurons and TNF-α specific marker cells in the ischemic cortex 72 h after FPI compared with the sham operation group (■) (P>0.05). Each column and bar is the mean±SD of eight rats per group.

**Figure 7:**
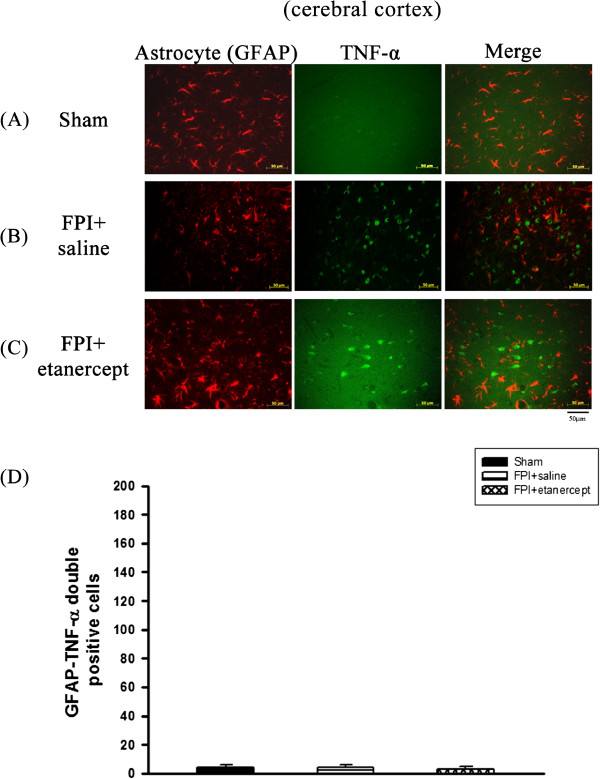
**Etanercept or FPI treatment did not affect the number of co**-**localization of astrocytes and TNF-α specific marker cells in the ischemic cerebral cortical regions.** Representative panel staining 72 h after sham operation or FPI, respectively for (**A**) a sham operation rat, (**B**) a FPI+saline rat (**C**) a FPI+etanercept rat. (**D**) The FPI+saline group (▤) or the FPI+etanercept group (▩) showed an insignificant change in the number of co-localization of astrocytes and TNF-α specific marker cells in the ischemic cortex 72 h after FPI compared with the sham operation group (■) (P>0.05). Each column and bar is the mean±SD of eight rats per group.

On the other hand, numbers of the co-localization of TNF-α and neurons specific marker cells (Figure 6A) or TNF-α and astrocytes specific marker cells (Figure 7) in the ischemic cortex of the saline-treated or the etanercept-treated FPI groups were not significantly different (P>0.01) from the sham group when evaluated 72 hours after FPI or sham operation.

### FPI caused overproduction of cerebral TNF-α, which etanercept attenuated

**Figure 8:**
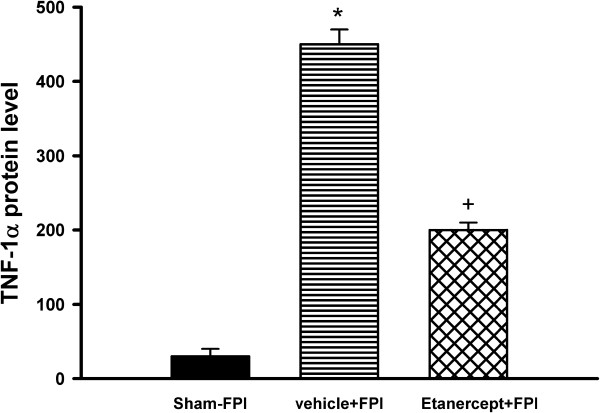
**Etanercept attenuated TBI**-**induced increased brain levels of TNF-α.** *The FPI+saline group (▤; n=8) showed a significant increase in ischemic cortical levels of TNF-α (P<0.01) compared with the untreated sham-FPI group (■). ^+^The FPI+etanercept group (▩; n=8) showed a significant decrease in the ischemic cortical levels of TNF-α (P<0.05) compared with the FPI+saline group (▤; n=8).

The cerebral levels of TNF-α were consistently significantly (P<0.05) higher for the FPI+saline and the FPI+etanercept groups than for the sham group 72 h after FPI or sham operation (Figure 8), but significantly lower in the FPI+etanercept group than in the FPI+saline group.

## Discussion

The major findings of our present study are: (1) etanercept injected systemically reduces motor and neurological deficits caused by FPI by day 3 after FPI; (2) the increased numbers of the co-localization of TNF-α and microglia specific marker cells are significantly and selectively higher in the ischemic cortex, white matter, hippocampus, and hypothalamus during FPI which can be attenuated by etanercept therapy; (3) overproduction of cerebral TNF-α in the ischemic cortex caused by FPI can be attenuated by etanercept; (4) by day 3 after FPI, neither the co-localization of TNF-α and astrocyte specific marker cells nor the TNF-α and neuron specific marker cells can be seem in the ischemic brain regions. Our data suggest that systemic administration of etanercept may improve outcome of FPI by attenuating the activation of microglial TNF-α in the ischemic brain. The hypothesis is in part supported by many investigations. For example, Li and Colleagues [16] has reported that TNF-α is significantly higher in the lesion boundary zone in the saline-treated rats by 3 days after FPI. Cerebral inflammation in response to trauma, stroke, and seizure is characterized by the activation of resident microglia [17-19]. Activated microglia proliferate, change morphology by assuming an amoeboid shape, increase phagocytosis, upregulate MHC class I molecules, and release cytokines [20,21].

TNF-α transduces death- and survival-signaling through its cognate receptors TNFR1 and TNFR2 and is involved in the inflammatory response following TBI [22-25]. Increases in TNF-α and other cytokines have been reported in cerebrospinal fluid and plasma samples in TBI patients [2,26-29]. Several groups [3,5,30-34] have reported increased TNF-α and other cytokine levels 1 h post-TBI, and peak levels 4 h post-TBI, after which, levels returned toward baseline. In Knoblach et al., [31], a secondary lesser increase at 72 h post-TBI was also reported. Furthermore, Holmin and Mathiesen [32] reported persistent elevations 4 days to 3 months after TBI, and Li et al., [16] found that TNF-α and other cytokines were significantly higher in the lesion boundary zone in saline-treated TBI rats 3 days post-TBI. In our rat model, microglial overproduction of TNF-α in several ischemic brain regions 3 days after TBI was also reported. In particular, Knoblch et al. [31] reported peak levels of TNF-α very early after TBI (1–4 hours) with localization to neurons, whereas our present results showed the peak levels of TNF-α occurred at 72 hours and were localized to the microglial cells. Thus, it appears that the cellular sources of this early elevation of TNF-α may be time-dependent. However, the most important point is that this elevated post-TBI TNF-α production in brain tissues can be significantly attenuated by etanercept therapy.

Accumulated evidence shows that TNF-α and its receptor play an important role in the pathophysiology of TBI [22,24,25]. In contrast, some evidence suggests that TNF-α plays a neuroprotective role following TBI [35,36]. Although TNF-α contributes to neuro-anatomical plasticity as well as an improvement of locomotor activity during recovery process [37], present data indicate that TNF-α is associated with the pathological effects as well as neurological motor deficits during acute phase after TBI. In the present study, despite the etanercept treatment there is still a robust TNF-α release post-injury. However, etanercept should be given only at acute phase [35,36]. A greater dose of etanercept administered during recovery process would not improve outcome and even exacerbate the pathological effects of TBI.

Etanercept, when administered systemically at the dosage approved for its licensed indications (~50 mg/week in human), would not be expected to achieved therapeutic levels in the cerebrospinal fluid because of its high molecular weight [38]. It should be mentioned that the etanercept doses used in the present set-up are far higher than the normal subcutaneous dose used for rheumatoid arthritis. Administration of a dose in humans might result in significant penetration of etanercept into the cerebrospinal fluid; particularly in an experimental setting such as TBI, in which the blood- cerebrospinal fluid barrier might be damaged.

The present study focuses mainly the effects of etanercept on microglial overproduction of TNF-α in the rat brain effected by TBI. Actually, in addition to TNF-α, TBI-induced increased levels of both interleukin-1β and interleukin-6 were all significantly reduced by etanercept treatment [8]. Additionally, Iba1 stain was chosen as a marker of activated microglia. Stains for surveillance microglia would help clarify if etanercept interferes with microglia activation and/or interferes with chemokines production and subsequent migration of microglia to the contused/ischemic areas of the brain. Furthermore, it is possible that the beneficial effects of etanercept during TBI are a result of limiting macrophage recruitment in part [39].

It should be mentioned that in the present study, there appears to be quite a few instances of Iba1 and TNF-α labeling that do not coincide and the staining morphology is quite different in some instances (mor elongated Iba1 staining versus round/amoeboid TNF-α staining). Furthermore, Iba1 is not a particularly distinguishing cell-specific marker for microglia in the injured brain as infiltrating macrophages also express this antigen. In addition, although our present study showed that the sham-group displayed no evidence of damage 3 days post-FPI, Jones et al. [12] did display evidence of damage one month post-FPI. The discrepancy between our results and their findings could be due to time difference.

Finally, it should be mentioned that given that the sham animals in the present study did not receive injections on any treatment the authors might comment on the possible confound that the stress associated with the injections may have had on the TBI group versus sham.

## Conclusion

The present study demonstrates that TBI, in addition to inducing cerebral contusion and neurological motor deficits, cause the microglial overproduction of TNF-α in the injured cortex, hippocampus, white matter, and hypothalamus. The TBI-induced neurological motor deficits and microglial overproduction of TNF-α can be significantly attenuated by the post-TBI application of etanercept. Our data identify a role for the microglial production of TNF-α in the outcomes of TBI in rats. Etanercept may attenuate cerebral contusion and neurological motor deficits during TBI by inhibiting the activation of the microglia-TNF-α double positive cells in the ischemic brain region.

## Competing interests

The authors declare that they have no competing interests.

## Authors’ contributions

CCC and CPC designed and conducted most of the experiments. CHC, CCW, CUC, CMC, BCC, CZY designed and conducted some of the experiments. CCC and CPC wrote most of the manuscript. All authors analyzed the data, revised the manuscript and gave final approval for publication.
